# V-Mango: a functional–structural model of mango tree growth, development and fruit production

**DOI:** 10.1093/aob/mcaa089

**Published:** 2020-07-18

**Authors:** Frédéric Boudon, Séverine Persello, Alexandra Jestin, Anne-Sarah Briand, Isabelle Grechi, Pierre Fernique, Yann Guédon, Mathieu Léchaudel, Pierre-Éric Lauri, Frédéric Normand

**Affiliations:** 1 CIRAD, UMR AGAP, *34098 Montpellier*, France; 2 AGAP, Univ Montpellier, CIRAD, INRAE, Institut Agro, *Montpellier*, France; 3 CIRAD, UPR HortSys, *97455 Saint-Pierre, La Réunion,*France; 4 HortSys, Univ Montpellier, CIRAD, *Montpellier*, France; 5 CIRAD, UMR QualiSud, *97130 Capesterre-Belle-Eau, Guadeloupe*, France; 6 Qualisud, Univ Montpellier, Avignon Université, CIRAD, Institut Agro, Université de La Réunion, *Montpellier*, France; 7 UMR ABSys, INRAE, CIRAD, CIHEAM-IAMM, Institut Agro, Univ Montpellier, *Montpellier*, France

**Keywords:** Mango tree, architecture, functional–structural plant model, generalized linear model, vegetative development, flowering, fruiting

## Abstract

**Background and Aims:**

Mango (*Mangifera indica* L.) is the fifth most widely produced fruit in the world. Its cultivation, mainly in tropical and sub-tropical regions, raises a number of issues such as the irregular fruit production across years, phenological asynchronisms that lead to long periods of pest and disease susceptibility, and the heterogeneity of fruit quality and maturity at harvest. To address these issues, we developed an integrative functional–structural plant model that synthesizes knowledge about the vegetative and reproductive development of the mango tree and opens up the possible simulation of cultivation practices.

**Methods:**

We designed a model of architectural development in order to precisely characterize the intricate developmental processes of the mango tree. The appearance of botanical entities was decomposed into elementary stochastic events describing occurrence, intensity and timing of development. These events were determined by structural (position and fate of botanical entities) and temporal (appearance dates) factors. Daily growth and development of growth units and inflorescences were modelled using empirical distributions and thermal time. Fruit growth was determined using an ecophysiological model that simulated carbon- and water-related processes at the fruiting branch scale.

**Key Results:**

The model simulates the dynamics of the population of growth units, inflorescences and fruits at the tree scale during a growing cycle. Modelling the effects of structural and temporal factors makes it possible to simulate satisfactorily the complex interplays between vegetative and reproductive development. The model allowed the characterization of the susceptibility of mango tree to pests and the investigatation of the influence of tree architecture on fruit growth.

**Conclusions:**

This integrative functional–structural model simulates mango tree vegetative and reproductive development over successive growing cycles, allowing a precise characterization of tree phenology and fruit growth and production. The next step is to integrate the effects of cultivation practices, such as pruning, into the model.

## INTRODUCTION

Improving the management of fruit trees implies a better knowledge of the impact of tree architecture on vegetative development and reproduction (Lauri, 2002; Costes *et al.*, 2006; Dambreville *et al.*, 2013*a*). Plant architecture, defined as the structural arrangement of plant organs in 3-D space (Godin, 2000), modulates internal physiological processes and is the interface of the plant with its environment. For instance, the spatial distribution of leaves determines light interception and carbon acquisition that, in turn, strongly affect the vegetative and reproductive growth of the tree. During tree ontogeny, the development of the tree architecture thus reflects the complex interplay between the structural organization and the spatial distribution of plant organs.

Plant architectural development can be formalized using the functional–structural plant model (FSPM) approach (Sievänen *et al.*, 2014). It has been successfully applied on fruit trees (Allen *et al.*, 2005; Lescourret *et al.*, 2011), forest trees (Letort *et al.*, 2008; Sievänen *et al.*, 2008), perennial grasses (Verdenal *et al.*, 2008) and annuals (Fournier and Andrieu, 1999; Buck-Sorlin *et al.*, 2008; Kahlen and Stützel, 2011; Barillot *et al.*, 2016; Louarn and Faverjon, 2018). This approach makes it possible to study *in silico* ecophysiological processes such as light interception (Da Silva *et al.*, 2014*a*) and carbon partitioning among the various plant organs (Génard *et al.*, 2008; Da Silva *et al.*, 2014*b*). It provides an easy means to generate a large number of similar trees (Han *et al.*, 2017), thus limiting the tedious work of data acquisition (Sinoquet *et al.*, 1997) despite the development of semi-automatic acquisition systems for plant geometry (Xu *et al.*, 2007; Boudon *et al.*, 2014; Hackenberg *et al.*, 2015). Exploring the model’ parameters space can help to design new ideotypes (Da Silva *et al.*, 2014*c*; Chen *et al.*, 2015; Perez *et al.*, 2018). It is also a step toward predictive tools for agronomy by integrating cultural practices such as pruning (Lopez *et al.*, 2010).

To simulate the complex morphogenesis of fruit trees, several studies have formalized the production of new organs using various stochastic models [e.g. hidden semi-Markov chain (HSMC) for modelling lateral productions at the node scale in Costes *et al.* (2008), or hidden variable-order Markov chains for modelling growth unit succession within sympodial trees in Costes and Guédon (2012)]. These stochastic models estimated from the data were used as empirical sub-models to simulate developmental patterns at different scales within FSPMs. These studies focus on temperate trees such as apple (Costes *et al.*, 2008) and peach (Lopez *et al.*, 2008) whose growth and development are markedly modulated by strong seasonality and a complete developmental reset during winter. Some first works addressed the problem of modelling tropical perennials. Perez *et al.* (2016) propose an architectural model of oil palm whose modelling benefits from the lack of ramification in its architecture. Closer to our work, Wang *et al.* (2018) propose a developmental model of annual growth modules of avocado over one growing season.

Our study addresses the problem of modelling complex architectural development of mango tree (*Mangifera indica* L.) over several growing seasons. Mango cultivation plays an important economic, nutritional and cultural role in tropical and sub-tropical regions, and its production ranks fifth in fruit production volume worldwide (Gerbaud, 2015). However, its cultivation raises a number of issues. In particular, its production is irregular from one year to the next, with strong heterogeneity of fruit size and gustatory quality at harvest. Moreover, phenological asynchronisms within and between trees (Normand and Lauri, 2018*a*) lead to long periods of critical phenological stages in the orchard that are susceptible to pests and diseases. Previous studies (Normand *et al.*, 2009; Dambreville *et al.*, 2013*a*) highlighted the importance of endogenous structural (i.e. position or fate of botanical entities) and temporal (i.e. their appearance dates) factors in the development of mango tree architecture. These factors were involved, for instance, in apical control (Normand *et al.*, 2009) or in the interplay between vegetative and reproductive growth (Dambreville *et al.*, 2013*a*). They may partially explain phenological asynchronisms and irregular bearing (Normand *et al.*, 2018). Modelling mango fruit production at the tree scale thus requires a detailed modelling of development of mango tree architecture and of its vegetative and reproductive organs. Our objective was to develop an integrative FSPM of mango tree development and fruit production based on current knowledge about vegetative and reproductive growth and development in order to: (1) demonstrate that an FSPM can be used to formalize the complex architectural development of evergreen tropical trees in terms of structure and phenology; (2) show that the introduction in the model of endogenous structural and temporal factors modulating tree architecture development allows the simulation of complex interactions between vegetative and reproductive growth; and (3) provide a tree growth and fruit production model representing the first step toward a mango crop model that would be used to design cultivation practices to alleviate agronomic issues.

We first provide some general information about mango tree architecture and development. We then introduce the modelling of the structure and development of the vegetative and reproductive organs, and then detail how they are assembled into a complete architectural model. Results on model parameterization and on the impact of endogenous structural and temporal factors on tree development and yield are presented and discussed. Finally, two applications of the model are presented. The first one is related to the characterization of the dynamics of phenological stages within a tree and an orchard, and the second one to the investigation of the effects of fruiting branch size on fruit growth and final mass.

## MANGO TREE STRUCTURE AND DEVELOPMENT

Mango development can be decomposed into growing cycles that last one and a half years (Dambreville *et al.*, 2013*a*; Normand and Lauri, 2018*b*). Each growing cycle consists of four main phenological periods. First, a period of about 9 months of vegetative growth takes place, during which 1–4 vegetative flushes generally occur on a tree. At the beginning of the cool and dry season, a resting period of about 2 months occurs and is followed by a 2–3 months flowering period. The fruiting period, that lasts approx. 4 months, is then composed of fruit growth up to harvest, which occurs at the beginning of the hot and rainy season. Since each cycle lasts >1 year, two successive cycles partly overlap on the same tree. The beginning of the vegetative growth period of a cycle overlaps with the flowering and fruiting periods of the previous cycle since vegetative growth can begin from the end of flowering.

**Fig. 1.**
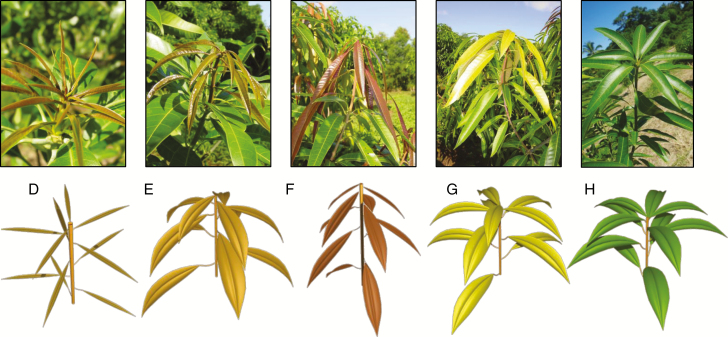
Observed and simulated phenological stages D (appearance of the axis), E (laminas half-opened starting to hang down), F (laminas totally opened hanging limply), G (leaves becoming rigid and moving upward) and H (mature growth unit) of the mango growth unit (stage nomenclature and top photographs from Dambreville *et al.*, 2015).

**Fig. 2.**
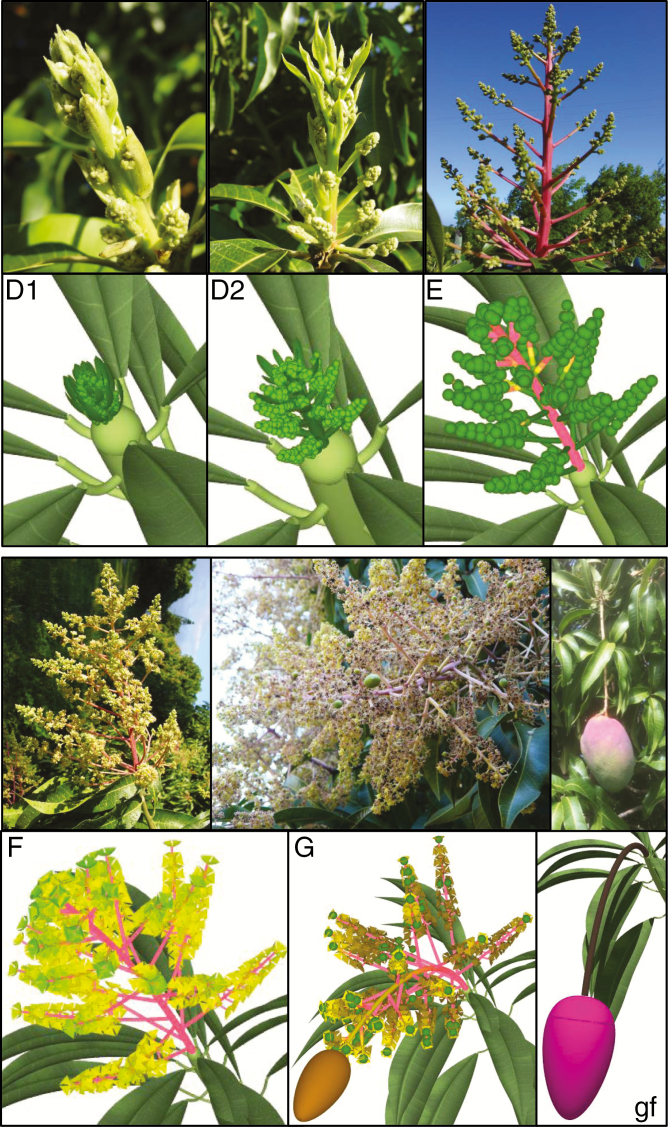
Observed and simulated phenological stages D1 (appearance of the main axis), D2 (bracts begin to fall), E (secondary axis moving away from main axis), F (flowering, from the first to last open flower), G (end of flowering) of the mango inflorescence, and growing fruit (gf) (stage nomenclature and photographs of stages D1–D2–E–F from Dambreville *et al.*, 2015; photographs for stage G and the growing fruit: F. Boudon).

The mango tree follows the architectural model of Scarrone (Hallé *et al.*, 1978), defined by a monopodial trunk bearing sympodial orthotropic branches with inflorescences in the terminal position. In this study, mango tree architecture was described as a collection of growth units, inflorescences and fruits organized into an arborescent structure. Mango vegetative growth is rhythmic and mainly sequential (Hallé *et al.*, 1978). Mango rhythmic growth produces growth units (GUs; Fig. 1), defined as the portion of an axis developed during an uninterrupted period of growth (Hallé *et al.*, 1978). The GUs are composed of a series of internodes and leaves arranged in a spiral and whose number is completely determined at bud burst. New GUs are positioned at the distal end of the previous ones, in either the apical or the lateral position, resulting in an acrotonic growth pattern. Herein, kinship terms are used for clarity, as proposed by Dambreville *et al.* (2013*a*). When a GU produces new GUs, the former is referred to as the mother GU and the latter are referred to as daughter GUs. The last GU developed during the vegetative growth period, which is in the terminal position and is able to flower and set fruit during the same growing cycle, is referred to as the ancestor GU. The GUs produced by an ancestor GU during the next cycle are referred to as descendant GUs. Flowers are borne by large highly branched inflorescences (Fig. 2). Some inflorescences can produce one to several fruits. Mixed inflorescences combine vegetative and reproductive traits: one to several leaves develop on the inflorescence axis, giving a leafy inflorescence (Davenport, 2009) usually without fruits. During their development, GUs and inflorescences go through a series of physical transformations including changes in size, orientation, texture or colour that allow characterization of the vegetative and reproductive phenological stages for the mango trees as described in Dambreville *et al.* (2015) and illustrated in Figs 1 and 2.

The study focused on the cultivar Cogshall. Data on mango tree architecture and on GU and inflorescence development were collected in Reunion Island (21°10'S, 55°50'E) and described in Normand *et al.* (2009) and Dambreville *et al*. (2013*a*, *b*). In a first experiment (dataset no. 1; Normand *et al.*, 2009; Dambreville *et al.*, 2013*a*), vegetative and reproductive development of three 3-year-old trees was exhaustively described during two consecutive growing cycles (cycles 1 and 2). All GUs, inflorescences and fruits appearing during the two cycles were recorded and described. The topological position (apical or lateral with respect to the mother GU) and the burst date (at the month scale) were recorded for each GU. The date of full bloom (at the week scale) was recorded for each inflorescence, and its date of burst was deduced using a thermal time model (see below). The harvest date (at the week scale) and the fresh mass at harvest were recorded for each fruit. In a second experiment (dataset no. 2; Dambreville *et al.*, 2013*b*), growth and development of a selection of GUs and inflorescences at the periphery of the canopy were recorded daily. The length of the main axis of the GUs, as well as the length and width of three individual leaves of the GU were measured from budburst to the end of organ growth. GU phenological stages were recorded from budburst to the end of organ development. These studies highlighted the importance of the effect of endogenous and environmental factors on the probability and date of burst, and on the growth and the development of GUs and inflorescences. In particular, structural and temporal factors influence the morphology and the period of appearance of GUs (Normand *et al.*, 2009; Dambreville *et al.*, 2013*a*). The rates of growth and development of GUs and inflorescences are affected by temperature (Dambreville *et al.*, 2013*b*).

## MODEL DESCRIPTION

### Overview of the V-Mango model

Mango tree architecture develops with flushes of GUs and inflorescences occurring over the growing cycle, whereas the growth of individual organs lasts from a few days to a few weeks for GUs and inflorescences, respectively (Dambreville *et al.*, 2013*b*). To integrate these different scales of development, we adopted a multiscale approach by formalizing several sub-models with different spatial and time scales, and combined them consistently.

First, a model of plant architecture development simulating the appearance of the different entities (GUs and inflorescences) in a tree over time was defined. To do this, we decomposed morphogenesis into elementary stochastic processes that simulate the occurrence, intensity and timing of development. These processes are first estimated/predicted at the month scale for the GUs and at the week scale for the inflorescences, consistent with the calibration data resolution. Then, a day is randomly chosen in the selected month or week to determine the precise burst date of each entity.

Sub-models then formalize the development and growth of the individual GUs, inflorescences and fruits at a daily scale. Growth and development of GUs and inflorescences were formalized using thermal time models. Fruit growth was simulated using the ecophysiological model proposed by Léchaudel *et al.* (2005, 2007). The inputs of these models were daily environmental data (temperature, relative humidity and solar radiation). In this section, we first describe the modelling of the structure and geometry of GUs, inflorescences and fruits, and of their growth and development. We then describe how these sub-models were assembled into an architectural model.

### Model of growth and development of growth units, inflorescences and fruits

#### Structure and geometry of growth units, inflorescences and fruits.

The use of plant models to provide realistic 3-D structures, in order to study light interception for example (Da Silva *et al.*, 2014*a*), requires precise representation of mango trees and their organs. We thus designed detailed models of the structure and geometry of GUs, inflorescences and fruits. GUs are described by several variables: axis length and diameter; number of leaves; individual leaf length, width and area; internode length; and phyllotaxy. The values of these variables were determined from empirical distributions measured on mature GUs and from relationships evidenced between them. These distributions and relationships were estimated from experimental data (dataset no. 2) and are presented in Table 1. As suggested by Normand *et al.* (2009) and Dambreville *et al.* (2013*a*), the influence of the position of the GU, apical or lateral with respect to the mother GU, and of the position of the mother GU with respect to its own mother GU were considered in the model when statistically relevant.

**Table 1.** Models for the variables characterizing the structure and geometry of mature growth units (GUs), mature inflorescences and growing fruits for the mango cultivar Cogshall

Botanical entity	Variable	Condition	Value	Equation number	Unit
Growth unit	Final length of GU axis (*L*)	Apical GU on apical mother GU	*N*(18.1, 4.1^2^)		cm
		Apical GU on lateral mother GU	*N*(13.8, 4.0^2^)		
		Lateral GU	*N*(12.6, 3.4^2^)		
	Number of leaves (*n*)	Apical GU	0.59 *L* + 5.50	(1)	–
		Lateral GU	0.62 *L* + 0.36	(2)	
	Final length of individual leaf (*l*)	Apical GU	*N*(17.1, 2.7^2^)		cm
		Lateral GU	*N*(14.9, 2.7^2^)		
	Leaf width		0.24 *l*	(3)	cm
	Leaf area		0.18 *l*^2^	(4)	cm^2^
	Final length of the first internode	Apical GU	Γ(2.0, 0.76)		cm
		Lateral GU	0.88 *L* + 0.38	(5)	
	Final length of internode at position *u*		*K* e^–2.64*u*^*L* with $K=\frac{\left(1-\sqrt[{n}]{e^{-2.64}}\right)}{\left(1-e^{-2.64}\right)}$	(6)	cm
	Basal diameter of the axis at the end of primary growth		*N*(0.025 *L* + 0.25, 0.11^2^)	(7)	cm
	Phyllotaxy (Goguey-Muethon, 1995)		144		°
Inflorescence	Final length of the main axis (*L*)		*N*(23.1, 6.7^2^)		cm
	Number of second-order axes		1.19 *L*	(8)	–
	Final length of second-order axes at position *v*		(0.69 *L* – 3.97)(1 – *v*)	(9)	cm
Fruit	Height		2.23 *M*_*f*_^0.29^	(10)	cm
	Largest diameter		1.25 *M*_*f*_^0.32^	(11)	cm
	Smallest diameter		0.98 *M*_*f*_^0.34^	(12)	cm

The main variables for growth unit and inflorescence models are the main axis length (*L*), and the fresh mass (*M*_*f*_) for the fruit model. Several traits of the growth unit model are conditioned by the apical or lateral position of the growth unit and possibly of its mother growth unit.

*N*, Gaussian distribution; Γ, Gamma distribution, *n*, number of internodes of the axis; *u*, normalized internode position along the main axis of the GU (*u* is equal to 0 for the second GU axis internode from the base, to 1 for the *n*th internode at the top, and to *i/n* for the *i*th internode from the base); *v*, normalized position along the main axis of the inflorescence (*v* is equal to 0 at the base of the inflorescence axis and to 1 at the top).

The first variable estimated by the GU model is the final length of the GU axis. It is of primary importance since other variables, the number of leaves, the final length of the internodes and basal diameter of the axis at the end of primary growth, are deduced from the GU axis length [Table 1; eqns (1), (2), (5), (6) and (7). The position of the GU and the position of the mother GU influenced GU axis length, leading to three statistically relevant distributions (Table 1). The number of leaves of the GU was calculated from the final GU axis length using a linear relationship whose parameters depended on the GU’s position [Table 1; eqns (1) and (2)]. The individual length of mature leaves followed a Gaussian distribution depending on the GU’s position. It was considered constant among the leaves of a GU, except for the two most distal leaves, which were smaller. Leaf length decrease was 52 % for the most distal leaf and 38 % for the penultimate leaf compared with the mean leaf length of the other leaves of the GU (Dambreville *et al.*, 2013*b*). The width and area of the leaves can then be determined from their length with the allometric relationships 3 and 4 given in Table 1. The length of the most basal internode, from the base of the GU to the basal leaf, was modelled differently for apical and lateral GUs because of the presence of a long basal internode on lateral GUs. It followed a gamma distribution for apical GUs, whereas it was linearly related to the GU axis length for lateral GUs [Table 1; eqn (5)]. Then, the length *L*_*u*_ of the other successive internodes *u* of the GU decreases more or less regularly from the base to the top of the axis [Table 1; eqn (6)]. This relationship was estimated using data from Goguey-Muethon (1995). The leaf arrangement along the GU axis followed a 2/5 spiral phyllotaxy (Goguey-Muethon, 1995). The basal diameter of the GU at the end of primary growth followed a normal distribution whose mean depends on axis length. Using these variables, the structure and the geometry of a mature GU can be precisely defined and represented (Fig. 1H; Supplementary data Information S1).

A similar approach was adopted to build a structure and geometry model for mature inflorescences. The final length of the main axis of inflorescences followed a Gaussian distribution. The number of second-order axes was linearly related to the main axis length [Table 1; eqn (8)]. The length of the second-order axes was modelled with a linearly decreasing function along the inflorescence main axis [Table 1; eqn (9)].

The fruit growth model proposed by Léchaudel *et al.* (2005, 2007) simulates fruit fresh mass every day (see below and Supplementary data Information S2). The shape of growing fruits was modelled as an ellipsoid whose dimensions, length and two diameters were determined from the fresh mass with allometric relationships [Table 1; eqns (10–12)].

#### Growth and development of growth units and inflorescences.

 Whereas the previous section focuses on describing mature GUs and inflorescences, in this section we examine how the considered variables changed over time to model growth and development. Growth duration and development of GU axes, leaves and inflorescence axes are strongly affected by temperature (Dambreville *et al.*, 2013*b*), justifying the use of thermal time models. These models consist of accumulating daily increments of development calculated as the difference between mean daily temperature and a base temperature *T* below which no development occurs. The growth duration or the phenological stage is completed when the sum of these daily increments (thermal time sum, *tts*) reaches a specific threshold *TTS*_*S*_ (Arnold, 1959; Bonhomme, 2000). Thermal time models therefore have two parameters, the base temperature *T* and the thermal time sum threshold *TTS*_*S*_. These parameters were estimated from dataset no. 2 from Dambreville *et al.* (2013*b*) for the main phenological stages of GUs and inflorescences (Table 2), and for the duration of growth of the GU axis, leaf and inflorescence axis (Table 3).

**Table 2.** Parameters of the thermal time models for each phenological stage of the growth units and inflorescences for the cultivar Cogshall

Botanical entity	Parameter	Phenological stages			
		D	E	F	G
Growth unit	Base temperature *T* (°C)	13.4	13.4	13.4	9.8
	Stage duration *TTS*_*S*_ (°Cd)	38.5	47.6	47.4	316.4
Inflorescence	Base temperature *T* (°C)	11.1	8.7	15.1	–
	Stage duration *TTS*_*S*_ (°Cd)	70.5	133.3	230.4	–

For the inflorescences, the phenological stage D combines stages D1 and D2 from Dambreville *et al.* (2015).

**Table 3:** Parameters for the sigmoidal growth models of the growth unit axes, leaves and inflorescence axes

Botanical entity	Base temperature *T* (°C)	Growth duration *TTS*_*S*_ (°Cd)	*t*_*ip*_ (°Cd)	*B* (°Cd)
Growth unit axis	9.2	178.8	89.4	22.4
Leaf	10.7	182.0	91.0	*L*/(0.06 *L*–0.08) (13)
Inflorescence axis	11.1	346.0	136.6	50.9

*t*_*ip*_ is the time of maximum growth rate and *B* is a slope parameter [see eqn (14)]. For the leaf growth model, the parameter *B* depends on the final leaf length *L*.

As suggested by Dambreville *et al.* (2013*b*), the growth of the GU axis, leaf and inflorescence axis was modelled using a sigmoidal curve (logistic function):

$$\begin{array}{l} l\left(tts\right)=\frac{L}{1+e^{-\frac{tts-t_{ip}}{B}}} \end{array}$$ (14)

where *l*(*tts*) is the entity length (cm) at thermal time *tts* (°Cd), *L* the final entity length (cm), *t*_*ip*_ the time of maximum growth rate (inflexion point of the curve, °Cd) and *B* a slope parameter (°Cd). Thermal time is counted from bud burst, i.e. phenological stage C for GUs and inflorescences (Figs 1 and 2; Dambreville *et al.*, 2015). In the modelling process, the final length *L* of GU axes, leaves and inflorescence axes were determined by the previous models of structure and geometry of these organs (Table 1). The growth curve parameters (*L*, *t*_*ip*_ and *B*) and the growth duration (*TTS*_*S*_) were estimated for each GU axis, leaf and inflorescence axis of the database of Dambreville *et al.* (2013*b*). Statistical analyses showed that *t*_*ip*_ could be estimated as half of the thermal time sum threshold required for growth duration for GU axes and leaves. This result did not hold for inflorescence axes, and the *t*_*ip*_ value for the inflorescence growth model was estimated as a constant equal to the mean of the estimated *t*_*ip*_ for each inflorescence. The slope parameter *B* was calculated from relationships between the maximal absolute growth rate at *t*_*ip*_ and the final length *L* (Dambreville *et al.*, 2013*b*). It was constant for the growth models for GU and inflorescence axes, and depended on the final length *L* for leaves [Table 3; eqn (13)].

The length of the internodes of a GU at a given time *tts* during growth was determined by applying the ratio between the current (at time *tts*) and the final length of the GU axis over their final length estimated as in Table 1, and eqns (5) and (6). A similar approach was used for GU diameter, leaf width and length of second-order inflorescence axes.

The GU secondary growth was modelled here using the pipe model theory (see Lehnebach *et al*., 2018 for a review). On the basis of this theory, a relationship can be established between the diameter *d* of a given GU and the diameters of its daughter GUs. We generalized it by considering the total number (*nbd*) of descendant GUs carried by a GU and established the following empirical relationship with its diameter *d:*

$$\begin{array}{l} d=\;0.88\;nbd^{0.41} \end{array}$$ (15)

During a simulation, the diameter *d* of a GU is updated each time a new descendant GU is produced.

#### Fruit growth.

The fruit growth model simulated the daily increase of fresh mass of each individual fruit. The fruit is assumed to go through two phases that are considered in the model: the first one corresponds to cell division and the second one to cell expansion. First, a dry mass *M*_*d*_ (g) is determined for each fruit at the end of the phase of cell division (352.7 °Cd after full bloom with 16 °C as the base temperature) by sampling in an empirical distribution modelled as a mixture of two Gaussian distributions (Léchaudel *et al*., 2006):

$$\begin{array}{l} M_{d}∼0.97\;N\left(13.9,\;4.1^{2}\right)+0.03\;N\left(29.2,\;0.66^{2}\right) \end{array}$$ (16)

The corresponding fruit fresh mass *M*_*f*_ (g) is then given by the following allometric equation:

$$\begin{array}{l} M_{f}=\;23.647\;*{M_{d}}^{0.6182} \end{array}$$ (17)

Secondly, the fruit growth model simulates growth during the cell expansion phase and determines the fruit fresh mass each day, as well as the final fruit fresh mass at maturity (Léchaudel *et al.*, 2005, 2007). It is based on a mathematical representation of carbon-related ecophysiological processes (i.e. leaf photosynthesis, mobilization/storage of reserves, respiration, demand for growth and carbon allocation) occurring at the fruiting branch scale (Léchaudel *et al.*, 2005), and water-related biophysical processes (i.e. water flows driven by stem and fruit water potentials and fruit transpiration) occurring at the fruit scale (Léchaudel *et al*., 2007). In this model, fruiting branches are assumed to be independent in terms of carbon balance. The model simulates changes in fruit dry and fresh mass at a daily time step, according to hourly weather conditions (temperature, relative humidity and light intensity), fruiting branch light environment and leaf to fruit ratio of the fruiting branch. The light environment of each fruiting branch is randomly selected from a set of contrasted environments, characterized by different gap fractions, measured in mango trees. The model also simulates the accumulation of organic compounds (sucrose, fructose, glucose, citric and malic acids) and minerals (K, Mg and Ca) in fruit flesh at a daily time step with empirical relationships between thermal time since full bloom and fruit flesh dry mass. A more detailed description of this fruit growth model is given in Supplementary data Information S2.

#### Graphical representations.

To graphically model development of GUs and inflorescences, specific sets of colours and branching angles for the leaves and second-order inflorescence axes were chosen for the different phenological stages of the GUs and inflorescences, respectively, and were linearly interpolated according to the development progress within the stage. Inflorescences also undergo a number of transformations. Flower buds were represented as simple spheres until they open. Individual flowers were then represented as simple polygonal meshes during the flowering stage F. Finally, to simulate the bending of the inflorescence axis under fruit mass, a simple gravitropism proportional to fruit fresh mass was applied. As a result, the image sequences depicted in Figs 1 and 2 could be generated.

### Model of tree architecture development

#### Description of the model of tree architecture development.

**Fig. 3.**
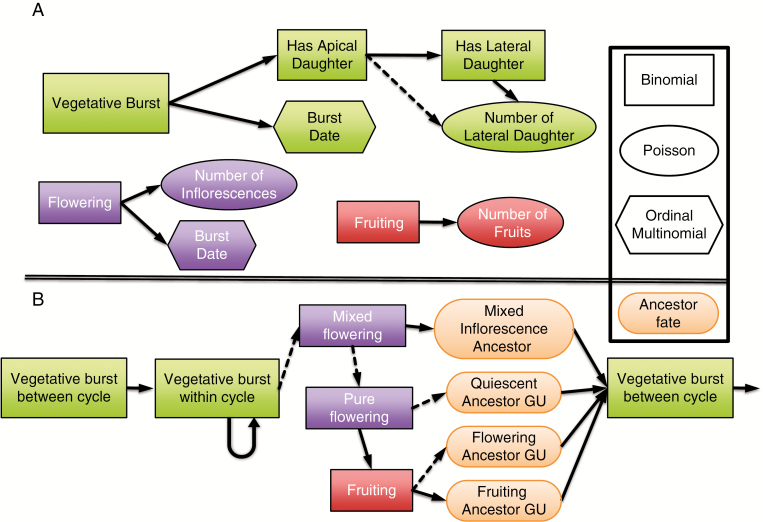
Stochastic automaton applied to each terminal growth unit (GU) to simulate the development of mango tree architecture and mango fruit production as directed graphs with nodes representing the different elementary developmental processes. The shape of each frame indicates the distribution underlying each developmental process or the ancestor fate entity in the case of pill-shaped frames. The edges indicate the succession of the processes. Their shape indicates that the succession is conditioned by a positive (solid arrow) or negative (dashed arrow) value of the realization of the parent binomial process. The three upper diagrams (inspired from Dambreville *et al.*, 2013*a*) represent vegetative (green automaton), flowering (purple automaton) and fruiting (red automaton) developments (A). The assembly of these automata into a complete developmental model is represented in the lower part (B).

The individual GUs, inflorescences and fruits, whose growth and development models are described in the previous section, need to be dynamically assembled into a complete architecture. Their appearance is considered at the scale of individual terminal GUs and can be decomposed into elementary processes describing the occurrence, intensity and timing of the development, modelled by binomial, Poisson and ordinal multinomial distributions, respectively (Dambreville *et al.*, 2013*a*). These processes are assembled to form vegetative burst, flowering and fruiting automata (Fig. 3A). Each automaton is a succession of stochastic processes driven by the value taken by the binomial distributions and determining the occurrence, the number and the appearance date of daughter GUs and inflorescences, and the occurrence of fruiting and number of fruits produced, respectively, on each terminal GU. These elementary processes are affected by structural and temporal architectural factors characterizing each terminal GU (Dambreville *et al.*, 2013*a*). The automata account for these effects by conditioning the statistical distributions underlying the processes with these factors (see below).

For vegetative burst, the production of at least one daughter GU is sampled for each terminal GU in a binomial distribution. If the answer is positive, a subsequent binomial distribution tests if there is one apical daughter GU. If not, we assume that all daughter GUs are in a lateral position. If there is an apical daughter GU, the automaton uses a specific binomial distribution to test if there is at least one lateral daughter GU. In the case of the occurrence of lateral daughter GUs, their number is determined with a Poisson distribution. Finally, the burst date of the daughter GUs is simulated with an ordinal multinomial distribution for which the month of bud burst is considered as the response, i.e. one of the 9 months of the vegetative period of the growing cycle. We assumed that all daughter GUs appear synchronously on a given terminal GU, which is the most common case.

GUs in a terminal position at the end of the vegetative period can potentially flower and set fruit. This is simulated by the flowering and fruiting automata, respectively. For each terminal GU, a binomial distribution tests if flowering occurs. If positive, the number of inflorescences is determined by a Poisson distribution, and the inflorescence(s) burst date (different weeks of the flowering period) is simulated by an ordinal multinomial distribution. Fruit set is then tested on flowering GUs using a binomial distribution. If positive, the number of fruits is sampled within a Poisson distribution.

These automata are then combined into the model of tree architecture development, an automaton at a higher level of abstraction that results from the chaining of the different vegetative, flowering and fruiting automata between and within growing cycles (Fig. 3B). The model starts at the beginning of the period of vegetative growth of the growing cycle, on the ancestor GUs, i.e. the terminal GUs at the end of the previous growing cycle. The automaton related to vegetative burst (Fig. 3A) is applied to the ancestor GUs to simulate the first layer of GUs during the current growing cycle (vegetative burst between cycles; Fig. 3B). The distinction between vegetative growth between cycles and within a cycle is justified by the fact that the ancestor GU is characterized by specific factors, in particular its fate, which affect its behaviour as a mother GU (Dambreville *et al.*, 2013*a*). The fate of the ancestor GU can be quiescent if the ancestor GU did not flower during the previous cycle, flowering if it flowered and did not set fruit, or fruiting if it flowered and set fruit. In this study, the factors that are considered to affect processes of vegetative burst between cycles are ancestor GU fate, date of burst and position. To initiate the simulation for each ancestor GU, values of these factors could be recorded on an actual tree, could be randomly sampled in empirical distributions or could result from the simulation of previous cycles (see below).

The automaton of vegetative burst is then applied on the GUs generated by the previous step (vegetative burst within a cycle, Fig. 3B). Factors considered to affect processes of vegetative burst within a cycle are mother GU position and date of burst, and ancestor GU position and fate.

When no vegetative burst occurs on a terminal GU during the vegetative growth period of a cycle, this GU is in the terminal position during the flowering period and is then susceptible to flower and set fruit (it becomes an ancestor GU for the following growing cycle). The model first tests if the GU produces mixed or pure inflorescences. We assume that only one type of inflorescence (mixed or pure) appears on a given GU. Using a binomial distribution, we first test if mixed inflorescences are produced. In the positive case, the number and the date of appearance of inflorescences are determined with a flowering automaton calibrated for mixed inflorescences. In the negative case, pure inflorescences are tested with a corresponding automaton. In the case of a pure inflorescence, the model then tests if the GU sets fruits with another binomial distribution. The fruiting automaton is then applied to simulate the number of fruits. In this study, the factors considered to affect the reproductive development processes are the position and the date of burst of the mother GU, and the fate and position of the ancestor GU of the previous cycle. The result of these simulations defines the fate of each ancestor GU for the simulation of vegetative growth during the following cycle (Fig. 3B). At the tree scale, the results of these simulations are used to calculate variables such as flowering rate, fruiting rate or fruit production.

Once the number of daughter botanical entities (GU, inflorescence, mixed inflorescence) and their burst date are simulated for each GU, buds representing these organs are positioned at the distal end of the GU axis, according to the phyllotactic angle of 144° with a branching angle of 60°. Fruits are distributed on the inflorescences of the fruiting GUs.

#### Model parameterization.

Parameters of processes of the automata were estimated individually on the basis of three measured Cogshall mango tree architectures presented in Dambreville *et al.* (2013*a*). In this context, vegetative growth occurs from September to May, flowering from July to September, and harvest from December to February. The database was converted into the MTG format (Godin and Caraglio, 1998). A Python script extracted the different GUs and the information related to their vegetative and reproductive development from this structural database. The resulting table was processed using R (R Core Team, 2016). The effects of the temporal and structural factors were tested with generalized linear models (GLMs), using the glm function for the binomial and Poisson responses, and the vglm function of the VGAM package (Yee, 2015) for the ordinal multinomial responses (Fig. 3). Significant factors were selected based on the Akaike information criterion (AIC) using the step function for the binomial and Poisson responses, and a specifically implemented function for the ordinal multinomial responses. Finally, the predict.glm and predict.vglm functions calculated the predicted probabilities for each combination of modalities of the significant factors. For each GLM, significant first-order interactions were integrated after the selection of the significant explanatory factors and were thus taken into account in the predicted probabilities estimation. All the probability tables were saved and used for the simulations.

**Fig. 4.**
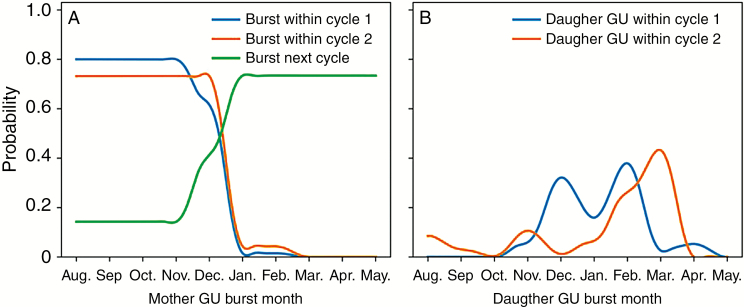
(A) Probability that a mother growth unit (GU) produces at least one daughter GU within the same cycle or during the following cycle (vegetative burst between cycles 1 and 2) according to its burst month. (B) Probability of appearance of daughter GUs for each month during cycles 1 and 2. These probabilities were inferred from measured development of three mango trees.

The probability tables determined with this parameterization method from empirical data highlighted several results that are presented below. For GU production within a growing cycle, GUs that burst from August to December had a high probability (*P *= 0.80) to produce daughter GUs within the same growing cycle, while those that burst later in the cycle had a low probability (*P *= 0.02) (Fig. 4A). As a result, GUs that appeared after December tended to produce their daughter GUs during the next cycle (*P *= 0.73).

GUs that produced daughter GUs within the same cycle had a high probability to produce an apical daughter GU (*P *= 0.98). Apical mother GUs had a higher probability to produce lateral daughter GUs (*P *= 0.66) than lateral mother GUs (*P *= 0.28) and, when they did, apical mother GUs produced a higher number of lateral daughter GUs than lateral mother GUs (on average, 2.1 vs. 1.3 for cycle 1, and 2.3 vs. 1.9 for cycle 2), in accordance with Normand *et al.* (2009).

During cycle 1, successive daughter GUs appeared with a regular pattern, on average 2.2 months after the burst date of the parent GUs, leading to regular peaks of GU production in December, February and April (Fig. 4B). During cycle 2 (Fig. 4B), the average delay between successive GU burst dates was about 3.0 months, leading to peaks of GU production in August, November and February/March. However, GU production was low from flowering to harvest (August to January) and high after harvest in February/March.

The production of GUs between growing cycles was strongly affected by the fate of the ancestor GUs. Only quiescent ancestor GUs could produce an apical daughter GU during the next growing cycle (*P *= 0.82) since the apical bud was transformed into an inflorescence for flowering and fruiting ancestor GUs. The probability of producing lateral daughter GUs for quiescent ancestor GUs was *P *= 0.46. The mean number of lateral daughter GUs depended on the fate and position of the ancestor GU. The flowering and fruiting ancestor GUs produced more lateral daughter GUs (3.4 and 4.2, respectively) than quiescent ancestor GUs (2.4), probably due to the loss of apical dominance with apical flowering (Normand *et al.*, 2009; Capelli *et al*., 2016).

**Fig. 5.**
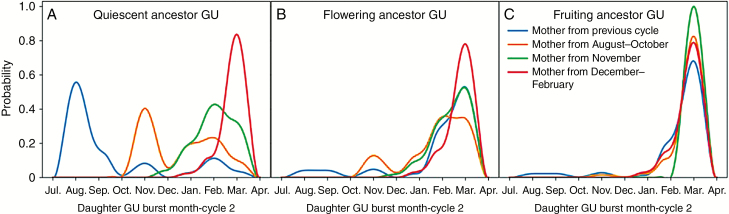
Monthly probability of the appearance of daughter growth units (GUs) during cycle 2 according to the burst month of the mother GU and the fate of the ancestor GU of cycle 1 [quiescent (A), flowering (B) or fruiting (C)]. The blue line represents direct daughter GUs of ancestor GUs (between-cycle production), and orange, green and red lines represent daughter GUs of mother GUs produced within the growing cycle (within-cycle production) during the periods of August to October, November, and December to February, respectively. These probabilities were inferred from measured development of three mango trees.

The fate of the ancestor GU also affected the timing of burst in the following cycle. Quiescent ancestor GUs tended to produce daughter and descendant GUs during the following cycle according to a 3 month pattern, depending on the ancestor GU burst date (Fig. 5A). Flowering (Fig. 5B) and fruiting (Fig. 5C) ancestor GUs produced daughter and descendant GUs mainly or only, respectively, after fruit production, and the ancestor GU burst date did not affect this pattern for fruiting ancestor GUs.

Mixed inflorescences were produced with a very low probability (*P *= 0.06) and mainly on terminal GUs generated between January and March. They were always the only inflorescence, located in the apical position. Mixed inflorescences themselves had a probability *P *= 0.53 to produce at least one daughter GU. They produced an average of 2.3 daughter GUs, and the probability that one of them was in the apical position was *P *= 0.47. Daughter GUs appeared on these mixed inflorescences between December and February.

Pure inflorescences were mainly produced by terminal GUs generated between October and May. The probability of flowering was positively affected by the apical position of the terminal GUs and by the non-fruiting fate of the ancestor GUs of the previous cycle (long-term effect of the ancestor GU fate). The number of inflorescences produced per terminal GU was usually one, except for GUs generated in December–January that produced an average of 1.6 inflorescences. The date of flowering of the inflorescences was recorded only during cycle 2, and most of the GUs flowered in September according to a two-peak pattern.

The fruiting probability was estimated at the terminal GU scale and not at the inflorescence scale. The date of flowering of the inflorescences affected the GU fruiting probability. The earlier the inflorescences flowered, the higher the GU fruiting probability was. The fruiting probability was *P *= 0.40 on average for GUs whose inflorescences flowered between July and 15 September, *P *= 0.20 for those whose inflorescences flowered from 15 September to 15 October, and 0 for those whose inflorescences flowered after 1 October. Apical GUs had a higher fruiting probability than lateral GUs. The number of fruits generated by a GU was positively related to the number of inflorescences of that GU.

#### Model implementation.

The implementation of the simulation model for plant architectural development was carried out using the L-Py module (Boudon *et al.*, 2012) of the OpenAlea platform (Pradal *et al.*, 2008) that mixes the formalism of L-systems (Prusinkiewicz, 1990) and the Python language. Simulations of the development and visual representation of the structure and its organs were thus formalized as L-system rules with the Python code. Rules related to the architectural sub-model are responsible for the creation of new botanical entities. Once created in the architecture, rules of the GU and inflorescence sub-models infer the attributes of the entities and simulate their development. The sub-model that formalizes fruit growth was developed with the R software (Léchaudel *et al.*, 2005). Communication between the main model in Python and this R sub-model was performed using file exchange or via the RPy 2 module. The main model provides parameters to the fruit growth sub-model such as the bloom date of the inflorescences and the number of leaves and fruits of the GUs belonging to the fruiting branches. In return, the sub-model provides the estimated dynamic of the fruits in term of fresh mass and organic compounds.

**Fig. 6.**
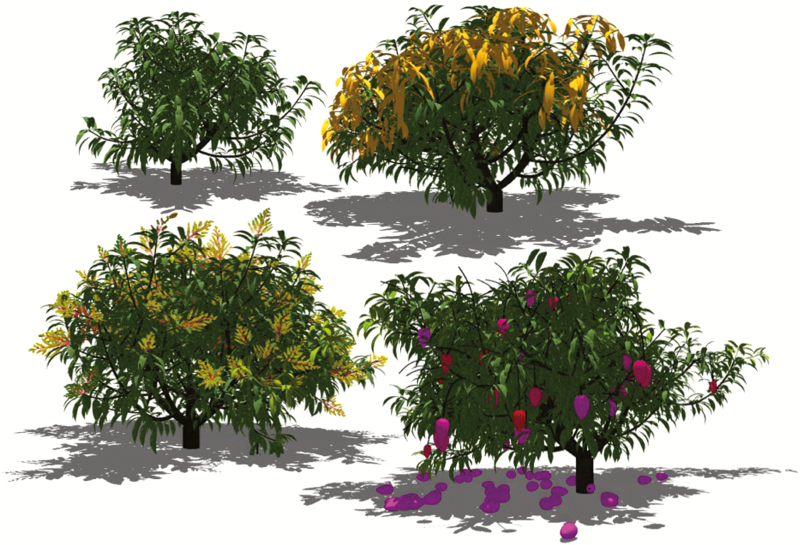
Stochastic simulation of the architecture of a mango tree at different periods of the growing cycle. From left to right and top to bottom, the initial structure, a flush of growth units during the period of vegetative growth (young extending leaves are in yellow), the flowering period with inflorescences and the fruit harvest period.

Finally, running the model makes it possible to achieve visual results such as those depicted in Fig. 6 and in Supplementary data Video S1.

## MODEL EVALUATION

The V-Mango model presented herein brings together numerous sub-models that act as local rules and are assembled as described above. It appears important therefore to evaluate how the model behaves as a complex assembly of components estimated individually. In order to perform this evaluation, we compared simulated data with data used for the parameterization at a global scale of a set of three trees during two growing cycles (dataset no. 1). The influence of structural and temporal factors on the architectural development and, consequently, their relevance to condition model probabilities was then quantified.

### Assessment of the global quality of the simulations

We used four global structural and temporal criteria, *C*_*i*_ i∊ [1,4], that were relevant to the objectives of the model, to evaluate the consistency of the assembly of the model components (Fig. 3). To assess the size and the dynamics of the population of botanical entities, we considered as global criteria the demography of the GUs (*C*_1_) and the demography of the inflorescences (*C*_2_), corresponding, respectively, to the number of GUs that burst each month and the number of inflorescences that bloomed each week during a simulated period. The criterion for fruit production (*C*_3_) was the total number of fruits produced per growing cycle. The fourth criterion was the distribution of axes lengths at the end of the two growing cycles (*C*_4_), a characteristic related to the organization of the generated architecture. In this case, an axis begins with a lateral GU and is formed by the successive apical GUs. The metrics is the number of GUs. We considered only the GUs that appeared during the simulated period.

These global structural and temporal criteria were computed on a set of three simulated trees and were compared with the values computed on a set of three actual trees. The starting point for simulations was the three trees described at the beginning of the first studied growing cycle. Their terminal GUs (=ancestor GUs) were individually characterized by their position and their fate. Vegetative growth, flowering, fruiting and fruit production were simulated during two successive growing cycles. One thousand simulations were performed and the four criteria *C*_*i*_ were computed for each simulation.

**Fig. 7.**
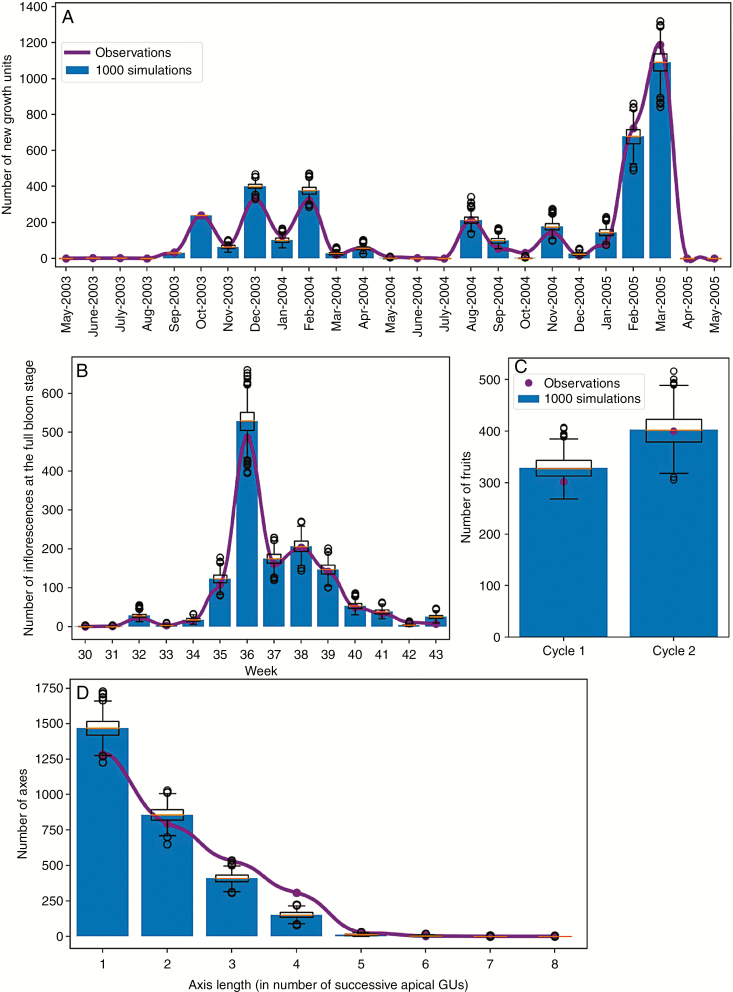
Global characterization of model simulation, with the blue histogram and boxplots representing, respectively, the average and distribution of 1000 simulations of three mango trees during two growing cycles. The purple lines and points are the actual values for the three measured mango trees. (A) The monthly demography of growth units (GUs) for growing cycles 1 and 2; (B) the weekly demography of inflorescences at the full bloom stage for the flowering period of cycle 2; (C) the number of fruits produced during each cycle; (D) the distribution of axis lengths produced during two growing cycles.

The results obtained for these four global criteria were in good visual agreement with the measured values (Fig. 7), the actual data being in the range of the simulated data. To assess this, we first computed an average value for each criterion from the 1000 simulations, thus representing an ‘average simulation’. For the GU demography, for instance, we computed the mean number of GUs generated each month from the 1000 simulations, resulting in a monthly mean number of GUs that appeared on the three trees during the two simulated growing cycles.

To test if the actual and the average simulated distributions were drawn from the same distribution, we performed a χ ^2^ test for each criterion. The *P*-values of the test for the demography of GUs and inflorescences were *P *= 0.24 and *P *= 0.23, respectively. It was *P *= 0.19 for the number of fruits produced and *P *= 0.22 for the axis length criteria. The hypothesis of dissimilarity was thus rejected, confirming the consistency between the actual and the average simulated distributions.

To characterize the error made by the simulations on each global criterion, we computed the root mean square error (RMSE) on the simulated values of each global criterion. RMSE for the criterion *C*_*i*_ was computed in the following way:

$$\begin{array}{l} RMSE_{i}(M,R)=\sqrt{\frac{1}{n}\sum_{k=1}^{n}{\left(r_{k}^{i}-v_{k}^{i}\right)}^{2}} \end{array}$$ (18)

where $r_{k}^{i}$ is the *k*-th component of the value for the criterion *C*_*i*_ estimated on the actual data *R*, and $v_{k}^{i}$ is the average value of the *k*-th component of *C*_*i*_ computed from 1000 simulations with the model *M*. In order to compare criteria, we normalized the root mean square error (NRMSE):

$$\begin{array}{l} NRMSE_{i}(M,R)=\;\frac{RMSE_{i}(M,R)}{\left(r_{max}^{i}-r_{min}^{i}\right)} \end{array}$$ (19)

where $r_{min}^{i}$ and $r_{max}^{i}$ are the minimum and maximum values of *C*_*i*_ for the measured data *R*, respectively.

In our simulations, NRMSE was about 3 % for the GUs and inflorescence demography, 4 % for the number of fruits and 7 % for axis length. This showed the good ability of the model to reproduce structures and dynamics similar to the actual ones.

### Influence of temporal and structural factors in architectural development

Similar to Dambreville *et al.* (2013*a*), the factors with a significant influence on each individual developmental process at the GU scale were identified using GLMs for each growing cycle (Table 4), and probability tables for each process in the model were determined accordingly. In a second step, a complementary analysis was conducted at the tree scale to quantitatively assess the influence of each factor on the simulation of the entire architectural development of a set of three trees. By comparing the analysis at the two scales, a better overview of the influence of the different factors could be assessed.

**Table 4.** Influence of the temporal and structural architectural factors (columns) on individual processes of vegetative and reproductive development at the growth unit (GU) scale (rows)

	Mother GU burst date	Mother GU position	Ancestor GU position	Ancestor GU fate
*Vegetative processes*				
Probability of vegetative burst	**	**	*	*
Probability to produce an apical daughter GU	*			
Probability to produce a lateral daughter GU	*	*		
Number of lateral daughter GUs	*	*	*	*
Date of vegetative burst	**			*
*Reproductive processes*				
Probability of flowering	**	**		**
Number of inflorescences produced per flowering GU	**	**		*
Date of burst of inflorescences	*			
Probability of fruiting	**	*		*
Number of fruits produced per fruiting GU		*		*

**The factor has a significant effect (*P*-value <0.01) on a process during both cycles and during transitions between cycles; its influence is considered as global. *The significant effect of the factor depends on the cycle and/or on the transition between cycles; its influence is considered as partial. No *, the factor has no effect on the process.

#### Characterization of the influence of the structural and temporal factors at the GU scale.

The significant structural and temporal factors for the different vegetative and reproductive processes are presented in Table 4. The burst date of the mother GU had a significant effect on all vegetative and reproductive processes, except the number of fruits produced per fruiting GU. The position of the mother GU and the fate of the ancestor GU also had a significant influence on most of the vegetative and reproductive processes. The influence of the ancestor GU position was limited to two vegetative processes (probability of vegetative burst and number of lateral daughter GUs).

However, since the number of processes in the model was high, with complex interdependencies, and the magnitude of the effect of each factor on the different processes varied considerably, it was not possible to assess the influence of each structural and temporal factor on the simulations at the tree scale with the complete model from these results at the GU scale.

#### Characterization of the influence of the structural and temporal factors at the tree scale.

To quantitatively assess the influence of each structural and temporal factor on the architectural development at the tree scale, we used the following methodology. First, a set of four new models was built, with each model *M*_*j*_, *j∊* [1,4], corresponding to the complete model *M*_*c*_ presented above without one of the structural or temporal factors *F*_*j*_ in the model parameterization. A null model *M*_0_ was also built without any factor. We then defined the influence index *I*(*C*_*i*_, *F*_*j*_), with *i∊* [1,4] and *j∊* [1,4], to measure the influence of a factor *F*_*j*_ on the global criteria *C*_*i*_:

$$\begin{array}{l} I\left(C_{i},F_{j}\right)=\frac{NRMSE_{i}\left(M_{j},M_{c}\right)}{NRMSE_{i}\left(M_{0},M_{c}\right)} \end{array}$$ (20)

The numerator represents the error made on the global criteria *C*_*i*_ by the simulations of a model *M*_*j*_ which did not consider the factor *F*_*j*_, compared with the error made by the complete model, thus giving a value for the benefit of taking into account the factor *F*_*j*_ in the complete model. To make the index comparable between all the criteria, the numerator was normalized by the NRMSE of the null model *M*_0_ compared with the complete model *M*_*c*_ for each criterion. One thousand simulations of a set of three trees were made to compute each index *I*(*C*_*i*_, *F*_*j*_).

A value of the index *I*(*C*_*i*_, *F*_*j*_) between 0 and 1 was expected, indicating the contribution of the factor *F*_*j*_ to the improvement of the simulations between the null model *M*_0_ and the complete model *M*_*c*_. The closer to 1 the index value was, the greater the contribution of the factor *F*_*j*_ was. A value above 1 indicated that the error made by model *M*_*j*_ was higher than that of the null model *M*_0_, and, thus, that considering only the factors other than *F*_*j*_ disrupted the simulation. The case *I*(*C*_*i*_, *F*_*j*_) >1 therefore confirmed the great importance of the factor *F*_*j*_ for controlling the structure and the temporality in the simulation.

The computed indexes *I*(*C*_*i*_, *F*_*j*_) are given in Table 5. The results showed that for the three global criteria related to the number and the temporality of the botanical entities in the structure (GU demography, inflorescence demography and number of fruits produced), a strong effect of mother GU burst date was observed, with influence indexes >1. The ancestor GU fate ranked second for its influence in the model. The other two factors seemed to have marginal effects on these three global criteria. For the fourth global criterion, axis length, related to the arrangement of the botanical entities, the mother GU position and burst date were the most important factors, with similar values of the influence index. The ancestor GU fate had an influence index value similar to the one it had for the three other criteria.

**Table 5.** Values of the influence index *I*(*C*_*i*_, *F*_*j*_) expressing the effect of each of the four temporal and structural architectural factors *F*_*j*_ (columns) used to condition the probabilities of the model of architectural development on the four global criteria *C*_*i*_ (rows) used to assess model simulation quality

Global criteria	Mother GU burst date	Mother GU position	Ancestor GU position	Ancestor GU fate
Growth unit (GU) demography	1.23	0.06	0.02	0.34
Inflorescence demography	1.46	0.11	0.10	0.18
Number of fruits produced	1.45	0.07	0.01	0.24
Axis length	0.82	0.83	0.04	0.28

## APPLICATIONS OF THE MODEL

Two examples of model applications are illustrated in this section. First, we show how a time series of susceptibility to pests can be estimated from the phenological modelling of each individual botanical entity of the tree. Secondly, we use an inverse modelling approach to determine the adequate size of fruiting branches to enable optimal fruit growth by comparing fruit size simulated by the model with different fruiting branch sizes with the actual fruit size. We thus assess their level of physiological autonomy (Sprugel, 1991) with regard to carbon supply to the fruit, which is a main aspect of fruit production sustainability (Lauri and Corelli-Grappadelli, 2014).

### Changes in tree phenology over time

**Fig. 8.**
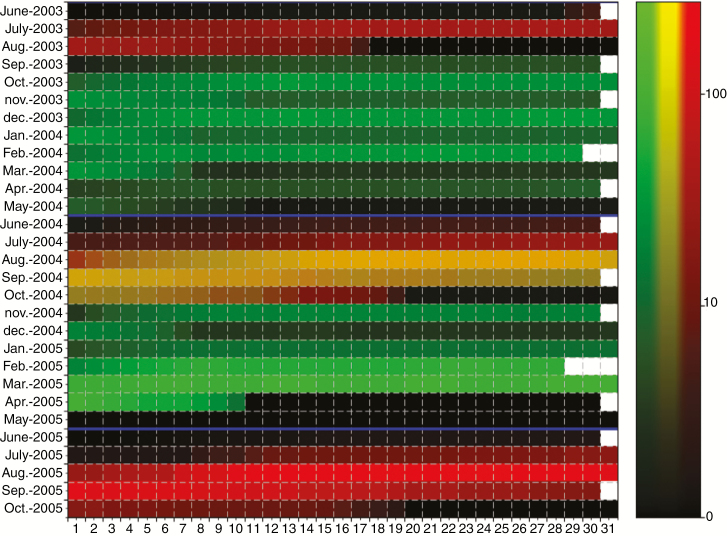
Daily simulated number of growth units (GUs, green channel), inflorescences (red channel) or both (in yellow) at phenological stage D or E per tree during two growing cycles. Months are in rows and days in columns. Data were computed from 100 simulated mango trees. Black colour indicates that no GU or inflorescence at stage D or E is simulated that day. The intensity is represented in log scale. Blue lines indicate the transition between two growing cycles (June).

Coupling of developmental models at the scale of the whole tree and at the scale of the individual botanical entities made it possible to precisely quantify the demography of the different phenological stages present at each date in a tree or an orchard. As an illustration, we estimated the daily number of GUs or inflorescences at the phenological stages D or E (Figs 1 and 2) during the two growing cycles of the simulation. These stages are known to be critical because GUs and particularly inflorescences at these stages are susceptible to the mango blossom gall midge, *Procontarinia mangiferae* (Felt) (Diptera: Cecidomyiidae), a pest of economic importance (Amouroux *et al.*, 2013). The model was run on a virtual orchard of 100 trees, each tree being randomly sampled within the three measured tree architectures. For each day of the two growing cycles, the number of GUs and inflorescences in the trees at phenological stages D or E were counted (Fig. 8). To improve visualization, these numbers were divided by the number of trees and thus give an average number of organs per tree.

The different peaks of production of the GUs and inflorescences are clearly identifiable on this diagram. For instance, the regular peaks of GU production in October and December 2003, February, August and November 2004, and February and March 2005 are visible, as well as the peaks of inflorescence production in July–September of each year. Concomitant production of GUs and inflorescences occurred in August and September 2004. In contrast, the periods without phenological stages susceptible to mango blossom gall midge, in general in May and June (in black in Fig. 8), illustrate the resting periods for the trees.

Such time series of susceptibility intensity can be a tool to study the interactions between pests and the mango orchard, and to optimize pesticide treatments or explore the effects of climate change scenarios on the susceptibility of an orchard to pests and diseases.

### Autonomy of fruiting branches

As a second application of our model, we explore the results from the coupling of the architectural model to the ecophysiological fruit growth model. This latter fruit model was originally used to evaluate the effects of the local leaf:fruit ratio, i.e. the respective size of carbohydrate sources and sinks during fruit growth at the branch level, on individual fruit mass at harvest. Fruiting branch autonomy for carbohydrates might depend on the size of fruiting branches, which defines the number of leaves and fruits and is therefore an important parameter (Léchaudel *et al.*, 2005). The objective of this model application was to assess the level of autonomy for carbohydrates of fruiting branches of different sizes in the mango tree.

**Fig. 9.**
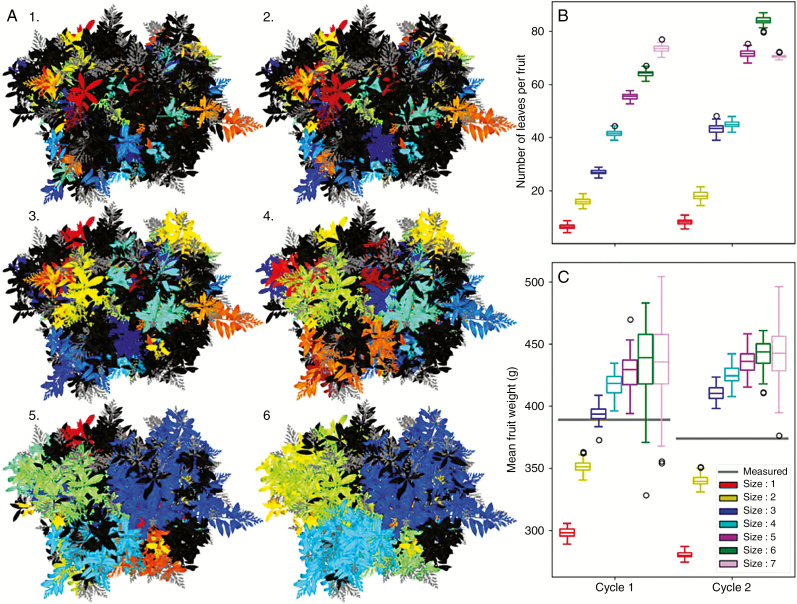
Testing the effects of different fruiting branch size on fruit growth. A fruiting branch of size *N* is defined as the set of GUs located at a maximal distance in the structure of *N* GUs from a fruiting GU. (A) Top views of a mango tree with fruiting branches of size 1–6. Different colours are used to identify the different fruiting branches. Non-fruiting inflorescences are in grey and growth units of non-fruiting branches are in black. (B) Distribution of the computed leaf:fruit ratio for each size of the fruiting branches during each simulated cycle. The leaf:fruit ratio is a main parameter controlling fruit growth. (C) The average fruit fresh mass simulated with different fruiting branch sizes compared with the measured ones (grey lines) during each simulated cycle. For graphs (B) and (C), boxplots represent the distribution of the average leaf:fruit ratio and of fruit fresh mass for 1000 simulations of three mango trees, respectively.

We first designed a parametric method to identify the fruiting branches in the simulated architectures. A fruiting branch of size *N* is defined as the set of GUs located at a maximal distance in the structure of *N* GUs from a fruiting GU. Considering the architecture as a tree graph of connected botanical entities (Godin and Caraglio, 1998), the distance between two entities is defined as the number of GUs making up the shortest path linking the two entities in the graph. This size *N* is a parameter of the model that can be controlled by the user. Practically, each fruiting GU was identified in the structure, and all the GUs that were at a maximal distance in the structure of *N* GUs from a fruiting GU belonged to the corresponding fruiting branch. If the sets of GUs of different fruiting branches overlapped, they were merged to form a unique fruiting branch with several competing fruiting GUs. Figure 9A illustrates how fruiting branches are distributed in the tree crown for different size *N*. With increasing branch size, GUs of the smallest fruiting branches are merged into fruiting branches of bigger sizes.

A sensitivity analysis of the effects of fruiting branch size *N* on the average fruit fresh mass at harvest was performed by testing small (*N *= 1, representing only the fruiting GUs) to large (*N *= 7 representing the scaffolds) fruiting branches. To make the results comparable, we fixed the development of the architecture by exactly reproducing the development of the measured trees. In this way, the number and timing of appearance of the organs were always the same. However, the morphology of the GUs, in particular, their number of leaves, was variable, simulated by the stochastic GU development model. Two successive growing cycles are considered. One thousand simulations with different random seeds were performed for each of the three measured trees. For each simulation, fruiting branches were identified and their leaf:fruit ratio was determined and used as input parameters in the fruit growth model to modulate carbohydrate acquisition from photosynthesis and partitioning between fruits according to their demand. The carbohydrate reserve in the fruiting branch at the beginning of fruit growth had a fixed value, independent of the fruiting branch size, taken from Léchaudel *et al.* (2005).

As a result, the leaf:fruit ratio linearly increased with the size of fruiting branches (Fig. 9B), ranging from ten to 90 leaves per fruit. However, the relationship between mean fruit fresh mass at harvest and fruiting branch size followed a sigmoidal pattern (Fig. 9C) with an asymptotic value of approx. 440 g during both cycles. The mean measured fruit fresh mass (390 g and 374 g for cycle 1 and 2, respectively) corresponded to the average fresh mass simulated with fruiting branches of size comprised between *N *= 2 and *N *= 3. This suggested a local effect of the architecture on fruit growth and, consequently, a relatively good autonomy for carbohydrates of the fruiting branches above size *N *= 3. This size of the fruiting branches is thus used in the model for subsequent simulations.

## DISCUSSION

We presented an FSPM of the architectural development and fruit production of the mango tree. At the GU scale, it is formalized as a stochastic automaton that decomposes the vegetative and reproductive development of the architecture into elementary processes modelled with probabilities estimated from actual trees using GLMs.

### GLMs provide a flexible modelling of developmental processes

From a methodological point of view, stochastic modelling of elementary developmental processes is flexible since it allows response variables following various distributions, i.e. binomial, Poisson and ordinal multinomial processes, in our model to represent occurrence, intensity and timing of a process, respectively. The distributions can be conditioned by various factors affecting the processes, after estimation with GLMs. These processes can then be assembled in a stochastic automaton representing a complex developmental model. This integrative approach that aims at modelling chains of causation in developmental processes is related to the approach of Pearl (2009). Thanks to the flexibility of the approach, the model of architectural development and fruit production can be enriched by taking other significant factors such as environmental conditions or cultivation practices into account, or by integrating other processes such as the death of botanical entities (Lauri, 2009). As a limit, however, increasing the number of factors exponentially increases the amount of required experimental data for model parameterisation.

This type of modelling is complementary to the hidden semi-Markov models integrated into other FSPMs (Costes *et al.*, 2008; Lopez *et al.*, 2008). Hidden semi-Markov models are two-scale models that can be used to model branching zones at the node scale (Guédon *et al.*, 2001; Costes *et al.*, 2008) as well as growth phases at the annual shoot scale (Guédon *et al.*, 2007). Different regression models can be incorporated into hidden semi-Markov models (see illustrations in Chaubert-Pereira *et al.*, 2009 and Lièvre *et al.*, 2016), including stochastic processes estimated with GLMs, as in our model.

### The development is modulated by structural and temporal factors

Consistent with the literature on mango (Dambreville *et al.*, 2013*a*) and more generally on fruit trees (Forshey *et al.*, 1989), the model takes into account some structural and temporal architectural factors to modulate the developmental processes.

#### Our method assesses the global impact of structural and temporal factors on model behaviour

We proposed the influence index as a method to quantitatively characterize the effect of each structural and temporal factor on the architectural development. This approach results from a multiscale analysis of the simulation (Godin, 2000; Dada and Mendes, 2011). While the factors conditioned the developmental probabilities at a local scale, their effects were characterized at the scale of the whole architecture, possibly over time. It is useful to validate or improve an FSPM by taking the global behaviour of the complex assembly of local rules into account and comparing the results with actual trees (Grimm *et al.*, 2005; Weinan, 2011). Such an approach can even be used in a reverse way to optimize the probability values according to the global characterization of the model.

#### Time is a major modulator of vegetative and reproductive development of the mango tree.

Our results revealed the major role of the temporal factor considered, the mother GU burst date, on the vegetative and reproductive development of mango tree architecture. Interestingly, this factor conditions probabilities in the simulation processes of growth and flowering of the mother GUs, and is itself a response of the simulation process for the daughter GUs. The rhythmic growth of mango trees, with regular delays between flushes (Fig. 5), seemed to be related to the effects of the mother GU burst date. Since GU and inflorescence burst dates (Fig. 3A) were important, they required precise modelling, justifying *a posteriori* their modelling as an ordinal multinomial distribution, and not as a simpler Poisson distribution to model the delay between mother GU and daughter GU burst dates, that led to less precise dynamics or demography of botanical entities (data not shown). While adapted to model the regular rhythmic growth observed, for example, during growing cycle 1, the Poisson distribution failed to simulate multiple flushes of daughter GUs for populations of mother GUs generated in the same month. It also failed to correctly model the synchronized vegetative flush occurring after fruit harvest during growing cycle 2 for mother/ancestor GUs generated in different months (Fig. 5C).

The ordinal multinomial distribution representing the different months of daughter GU burst date involved a large number of parameters, particularly if conditioning factors such as ancestor GU fate or mother GU burst date were considered. This large number of parameters allowed a better understanding and more precise simulation of the dynamics of the mango tree development. However, a large database was then required to fit the model, and possible overfitting might occur. An alternative, mechanistic and more parsimonious approach would require more precise identification of the endogenous mechanisms and environmental factors affecting bud burst, and their modelling.

### Reproductive growth during one cycle affects vegetative and reproductive growth during the following cycle.

The vegetative or reproductive fate of the ancestor GU affected the vegetative production as well as the reproduction during the following cycle, indicating long-term and long-distance effects of this factor (Dambreville *et al.*, 2013*a*). While descendant GUs of quiescent ancestor GUs followed a regular delay between vegetative growth events, the descendant GUs of flowering and fruiting ancestor GUs burst mostly, or only, respectively, after fruit harvest (Fig. 5). Ancestor GU fate thus seemed to contribute to the vegetative asynchronisms observed in mango tree development (Dambreville *et al.*, 2013*a*).

### Apical control modulates branch architecture.

We characterized the influence of the mother GU position, especially in the structuring of the architecture, which was assessed on the basis of axis length distribution (Table 5). The distribution and, in particular, the ratio between short and long axes that can be deduced from these data may be the signature of the promotion of certain GU types. The influence of this factor could be related to the role of the apical control in the architecture edification, which favours apical daughter GUs over lateral ones. There is debate in the literature on the mechanisms on which apical control relies (Wilson, 2000). In particular, nutrient retention or auxin production by the apical bud repressing lateral bud development are hypothesized (Cline *et al.*, 2009). The sensitivity analysis of our model suggested that this control was a short-distance control since the mother GU position had a large influence on axis length distribution, whereas the ancestor GU position had a very limited influence (Table 5). It was therefore restricted to the terminal growing parts of the structure in the mango tree.

### Toward cultivation practices

Using the V-Mango model, two applications have been developed that make a step toward the virtual design of cultivation practices.

#### Simulating detailed distribution of phenological stages leads to practical applications.

The V-Mango model includes thermal time sub-models that simulate the growth and development of individual GUs and inflorescences in response to temperature. They simulate the stage of development of the GUs and inflorescences at each date (Fig. 8).

The burst date of each individual GU was simulated at the month level by the model of architectural development, and a burst date was then randomly selected within the month days. The model did not account for possible peaks of co-ordinated GU production, and periods of vegetative growth may be artificially extended. The model needs to be refined to better integrate the dynamics of GU burst, thus making it possible to better characterize the duration of periods with organs at stages susceptible to pests and diseases.

The detailed characterization of the phenological stages existing within a tree or an orchard can be used for the coupling of V-Mango with models of pest or disease propagation and damage. It is also a basis to study the effects of climate change on mango phenology and production (Normand *et al.*, 2015).

#### Sensitivity analysis suggests a partial autonomy of fruiting branches.

The ecophysiological fruit growth model in V-Mango also made it possible to investigate a local effect of tree architecture on fruit growth. Indeed, simulations suggested that a fruiting branch composed of GUs at a distance of two to three GUs from the fruits had sufficient leaf:fruit ratio and reserves to feed them with photosynthates. These results are supported by those of Grechi and Normand (2019). The fruit model was originally calibrated for girdled fruiting branches, i.e. branches that were independent for carbohydrates, since girdling prevents phloem flux between the branch and the rest of the tree. Consequently, the model assumes independence between fruiting branches for carbohydrates, even if it is applied on trees with non-girdled branches.

However, it has been shown that leaf and stem carbohydrate reserves decrease during fruit growth in girdled fruiting branches, regardless of the leaf:fruit ratio (Léchaudel *et al.*, 2005), and in other compartments of the mango tree such as the main branches and roots (Stassen and Janse van Vuuren, 1997). These results suggest that (1) photosynthesis is not sufficient to fully support fruit growth and (2) long-distance carbohydrate exchange occurs in the mango tree, and it probably increases under constrained conditions (heavy fruit load, unfavourable environmental conditions).

An improvement of the model would thus be to integrate carbohydrate exchange between fruiting branches and other parts of the tree, similarly to other FSPMs such as L-Peach (Allen *et al.*, 2005) or QualiTree (Lescourret *et al.*, 2011). As girdling on fruiting branches, with growing fruits as active carbohydrates sinks, may have stimulated photosynthesis to compensate for exchanges between branches, a partial recalibration of the fruit growth sub-model may be necessary.

Another improvement of V-Mango is to integrate a light interception module to better take into account the light availability on each individual leaf according to the 3-D arrangement of the canopy and the foliage. This would give more accurate input for photosynthesis estimation in fruit growth simulation. Moreover, the mango tree carbohydrate reserves that can be mobilized for fruit growth at the different scales of the architecture hierarchy must be further investigated (Stassen and Janse van Vuuren, 1997).

As a perspective, the identification of the size of fruiting branches opens up the possibility to explore their structural configurations in a mango tree architecture and could help in identifying favourable and unfavourable conditions for fruit growth within a tree to optimize mango yield.

### Conclusion

The FSPM V-Mango presented herein is based on the modelling of the vegetative and reproductive development of the mango tree and their interactions. To our knowledge, such a perennial tropical fruit tree FSPM with the modelling of the development of a complex architecture with a large degree of ramification over several growing seasons is unique. It allows a didactic visual representation and exploration of the growth and development of the mango tree and its organs. It constitutes a basis for the development of agronomic tools to design cultivation practices aiming at maximizing and making more regular mango yield, and reducing pesticide use. It is also a basis for *in silico* ecophysiological experiments to be further validated in field trials. The model is currently parameterized with data from young unpruned mango trees (4–5 years old). Information on the development of adult trees should be integrated since they tend to produce fewer vegetative flushes during each cycle. GU mortality and leaf shedding should also be integrated. Finally, to make this model a useful tool for agronomy, the effects of cultivation practices such as pruning should be integrated, as well as interactions with pests.

## SUPPLEMENTARY DATA

Supplementary data are available online at https://academic.oup.com/aob and consist of the following. Information S1: visual representation of the 3-D model of GU and its different parameters. Information S2: summary of the fruit growth model. Video S1: animation representing the development of a mango tree during two growing cycles.

mcaa089_suppl_Supplementary_Video

mcaa089_suppl_Supplementary_Figure

mcaa089_suppl_Supplementary_Information_S2
