# Supplementary figures and images for: V-Mango: a functional–structural model of mango tree growth, development and fruit production
Source: Ann Bot. 2020 Jul 18;126(4):745–63. doi: 10.1093/aob/mcaa089 (PMC7489065; doi:10.1093/aob/mcaa089)

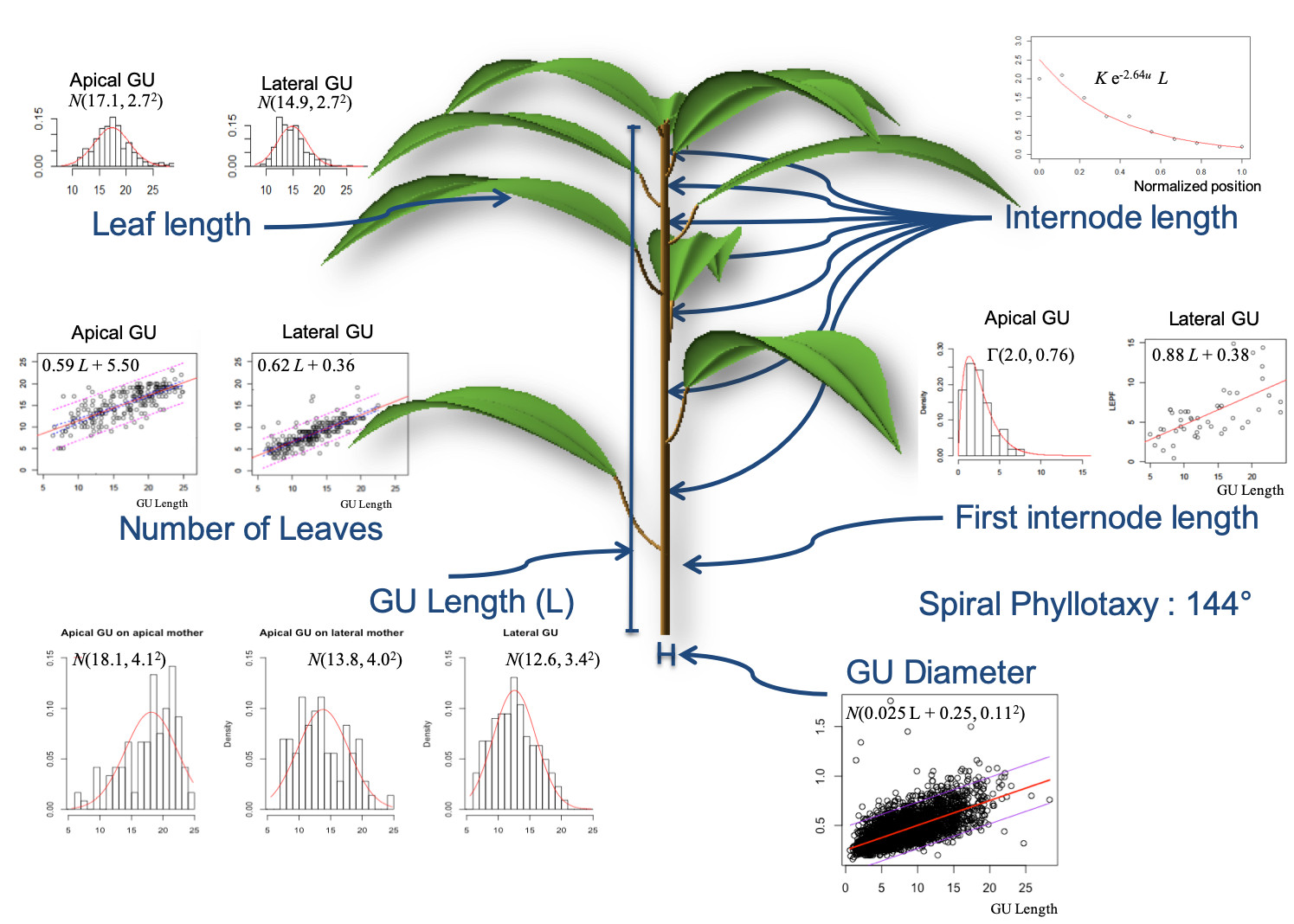

Supplement: mcaa089_suppl_Supplementary_Figure [file mcaa089_suppl_supplementary_figure.jpeg]
