# Supplementary material for: V-Mango: a functional–structural model of mango tree growth, development and fruit production
Source: Ann Bot. 2020 Jul 18;126(4):745–63. doi: 10.1093/aob/mcaa089 (PMC7489065; doi:10.1093/aob/mcaa089)
Supplement: mcaa089_suppl_Supplementary_Information_S2 [file mcaa089_suppl_supplementary_information_s2.docx]

**Supplementary Information 2: Mango fruit growth model presentation**

The model of mango fruit growth in dry mass and fresh mass runs on a daily time step at the scale of the fruiting branch. Experiments and model equations are described in Léchaudel et al. (2005) and Léchaudel et al. (2007).

The model combines two sub-models which describe the management (i) of carbon and (ii) the accumulation of water and sugars, acids and mineral compounds in the fruit. The carbon sub-model simulates changes in the carbon mass of the various compartments of the fruiting branch that are later transform in dry mass. A general view of the model is given in Fig. 1 and the equations governing the different sub-models are described below. The various sub-models were calibrated using experiments on girdled fruiting branches in order to control leaf-to-fruit ratios and reserves within fruiting branches.


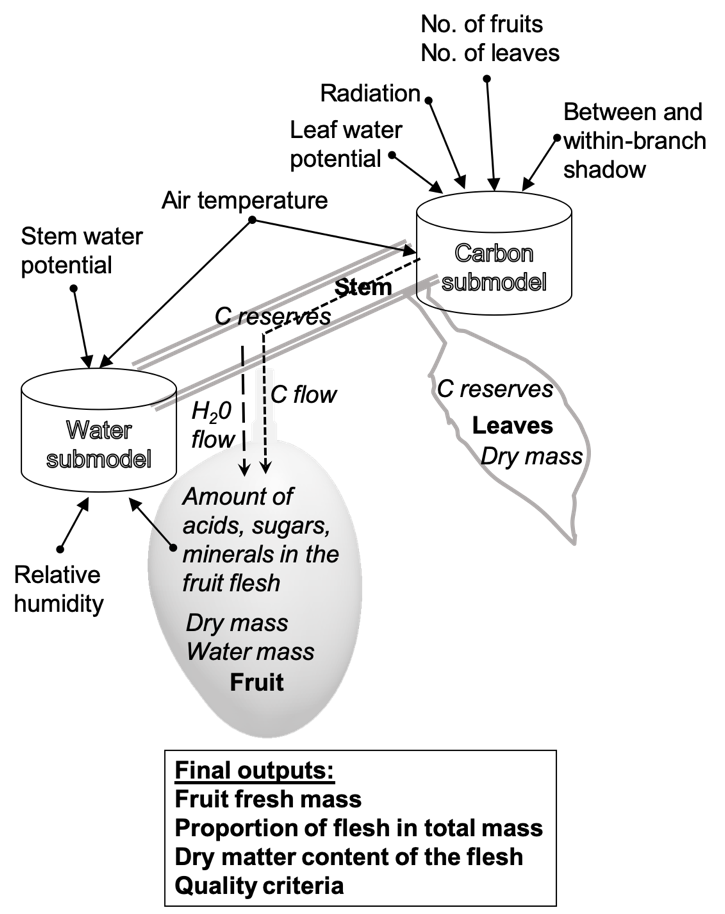


Figure 1: A visual representation of the fruit growth model (inputs = arrows with point)

***Sub-model of carbon balance:***

The carbon balance sub-model at the fruiting branch scale has been described by Léchaudel et al. (2005). It has been successfully tested with data sets corresponding to fruit growth on girdled fruiting branches with contrasted leaf-to-fruit ratios (10 to 150 leaves per fruit) on three different years.

The mango fruiting branch is divided into three compartments: fruits, stem and leaves. The pool of carbon available daily within the fruiting branch results from carbon assimilation by leaves (photosynthesis) and carbon mobilization from reserves of stem and leaves. Leaves photosynthesis, *P_l_*, is modelled as a function of the fruit demand $D_{fruit}$ (see below) and the photosynthetic photon flux *PPF*, with an asymptotic value $P_{max}^{*}$ (in μmol CO_2_ m^–2^s^–1^)

$P_{l}=\left( P_{max}+p_{3} \right)\left( 1-\exp\left( \frac{-p_{4}\cdot PPF}{P_{max}+p_{3}} \right) \right)-p_{3}$ (1)

$P_{max}=min(P_{max}^{*},\frac{p_{1}{\cdot D}_{fruit}p_{2}}{p_{1}\cdot D_{fruit}{+p}_{2}})$ (2)

with $p_{1}$to $p_{4}$ parameters of the photosynthesis function estimated experimentally.

Shaded and sunlit leaves were considered. The fraction of sunlit and shaded leaf area within the fruiting branch was estimated experimentally from fish-eye photographs and was integrated over the sun path. Net photosynthesis of shaded leaves was modulated by decreased radiation obtained with an empirical linear relationship with PPF.

Total daily amount of carbon *C_l_* fixed by leaf photosynthesis (g C.d^-1^) is determined as the sum of hourly photosynthesis of the shaded plus sunlit leaf area.

$C_{l}=k\cdot(\sum P_{l}^{sunlit}\cdot{LA}_{sunlit}+\sum P_{l}^{shaded}{\cdot LA}_{shaded})$ (3)

with *LA_sunlit_* and *LA_shaded_* the total area (m^2^) of sunlit and shaded leaves, respectively, and *k*=0.0432 the conversion coefficient of leaf photosynthesis from μmol CO^2^ s^–1^ to g C h^–1^. The leaf area *LA* of sunlit (resp. shaded) leaves is estimated from the number of sunlit (resp. shaded) leaves *n_l_* in the fruiting branch.

$LA=0.0051 \cdot n_{l}^{0.937}$ (4)

Carbon is allocated according to organ and priority rules: maintenance respiration costs of the three compartments are given first priority and fruit growth is given second priority. The assimilates unused for maintenance and fruit growth accumulate in the reserve pool of leaves and stem. Daily carbon demand, $D_{fruit}$, for fruit growth is function of the potential fruit growth rate $\frac{d\left( {DM}_{f}^{pot} \right)}{d\left( dd \right)}$, the carbon concentration (*c_fruit_*) in the fruit, and the growth respiration coefficient (*GRC_fruit_*). The daily potential growth rate of the individual fruit is modeled as:

$\frac{d\left( {DM}_{f}^{pot} \right)}{d\left( dd \right)}={RGR}_{f}^{ini}\cdot{DM}_{f}\cdot\left( 1-\frac{{DM}_{f}}{{DM}_{f}^{max}} \right)$ (5)

with $d\left( dd \right)$, the daily variation of degree days (*dd*), ${RGR}_{f}^{ini}$, the initial relative fruit growth rate (*dd^-1^*), ${DM}_{f}$, the fruit dry mass (g), i.e. the sink size, and ${DM}_{f}^{max}$, the maximal final dry mass (g).

Dry mass partitioning between the flesh and the stone within the fruit is derived from an empirical relationship between stone dry mass and total fruit dry mass.

***Sub-model of water balance and biochemical compounds:***

The water sub-model is an adaptation of the biophysical model of elastic and plastic fruit growth due to water developed by Léchaudel et al. (2007). We use a daily resolution scheme that allows to simulate only plastic growth due to water accumulation. The daily rate of change in water mass accumulated in the fruit flesh (${dw}/{dt}$) is calculated assuming that water enters the fruit flesh from xylem (U) and phloem (P) and is lost through fruit transpiration ($T_{f}$):

$\frac{dw}{dt}=U+P-T_{f}$ (6)

$T_{f}$, is calculated according to Fishman and Génard (1998) as a function of relative humidity difference between the fruit (*H_f_*) and the air (*H_a_*), the fruit area and the surface conductance of the fruit peel (*ρ*).

Phloem fluxes are estimated as

$P= \frac{1}{D_{s}}\cdot\frac{d(MS)}{dt}$ (7)

with $D_{s}$ the density of flesh dry matter.

Xylem fluxes are estimated as

$U=A\cdot L_{f}\cdot\left( \Psi_{Stem}-\Psi_{Fruit} \right)=A_{f}\cdot a.L_{f}\cdot\left( \Psi_{Stem}-\Psi_{Fruit} \right)$ (8)

where $A$ is the external area of the vascular network, assumed to be proportional to fruit area $A_{f}$ and a coefficient *a*, $L_{f}$ (g cm^-2^ MPa^-1^ d^-1^) is the hydraulic conductivity between the stem and the fruit including that of xylem and that of phloem, $\Psi_{Stem}$ (MPa) is the stem water potential and $\Psi_{Fruit}$ (MPa) is the fruit water potential, calculated as

$\Psi_{Fruit}=-\pi_{f}+P_{f}$ (9)

with $\pi_{f}$ (MPa) is the osmotic pressure in the fruit and $P_{f}$ (MPa) is the turgor pressure.

The osmotic pressure is obtained by the following equation, according to the definition of Van’t Hoff:

$\pi_{f}= \frac{R\cdot T\cdot n_{s}}{v}$ (10)

where $n_{s}$ is the number of moles of osmotically-active solute, *T* is the temperature of the air and $v$ is the volume of water in the flesh (cm^3^) estimated from the flesh water mass *w* and R the universal gas constant 8.314 (J mol^-1^K^-1^).

An empirical biochemical compounds sub-model was developed to simulate the various osmotically active solutes that contribute to the osmotic pressure (i.e., sugars, organic acids, minerals and amino acids). These compounds were individually expressed in the model, except for amino acids, which were considered as a global pool. The total number of moles of osmotically active solutes is:

$n_{s}= \sum_{j} n_{j}+n_{aa}$ (11)

where $n_{j}$ is the number of moles of the osmotically active solute j (i.e., individual sugars (sucrose, glucose, fructose), organic acids (citric, malic, oxalic, pyruvic) and minerals (calcium, magnesium, potassium, sodium, ammonium) in ‘Cogshall’ mango flesh, and $n_{aa}$ is the number of moles of all amino acids.

The number of moles of osmotically-active solutes j was expressed for each one as:

$n_{j}= \frac{prop_{j}\cdot DM}{MM_{j}}$ (12)

where ${prop}_{j}$ (dimensionless) is the mass proportion of the osmotically-active solute *j* in the flesh dry mass ($DM$, g) and ${MM}_{j}$ (g mole^-1^) is the molar mass of the osmotically-active solute *j*.

The proportion of osmotically-active solute *j* in the flesh dry mass was estimated statistically by linear regression with the following explanatory variables: the sum of degree days after full bloom, the dry mass of flesh and their interaction. During the simulation of fruit growth, the dry mass of the flesh is calculated by the carbon balance sub-model.

The turgor pressure of the fruit flesh, $P_{f}$, is calculated by solving the Lockhart equation describing the growth in volume, *V*, of the fruit flesh (Lockhart 1965) as a function of pressure and current fresh mass:

$\frac{dV}{dt}=\phi\cdot V\cdot\left( P_{f}-Y \right) if \left( P_{f}>Y \right)$ (13)

$$\frac{dV}{dt}=0 if \left( P_{f}\leq Y \right)$$

where *ɸ* is cell walls extensibility and *Y* is the pressure threshold value that the turgor pressure of the fruit flesh has to exceed before irreversible expansion occurs. *ɸ* is supposed to decrease over time and is thus defined as *φ_max_* before an initial time *dd_ini_* and then using a decreasing rate *τ*. Conversely, *Y* increases over time proportionally to the increase in fruit volume with a ratio *h*.

Assuming that the change in fruit flesh volume mainly results from water balance, it can also be calculated that:

$\frac{dV}{dt}=\frac{U+P-T_{f}}{D_{w}}$ (14)

where $D_{w}$ is the water density equal to *1*.

Under the condition of steady irreversible growth, it results from equations (13) and (14) that

$$\phi\cdot V\cdot\left( P_{f}-Y \right)=\frac{U+P-T_{f}}{D_{w}}$$

and

$$P_{f}=Y+\frac{U+P-T_{f}}{D_{w}.\phi\cdot V}$$

**Model integration and parameters**

The fruit growth model takes as input the number of leaves *n_l_* and fruits *n_f_* of the fruiting branch and the inflorescences blooming date D_0_, provided by the sub-model of architectural development, and environmental data such as daily average temperature *T*, relative humidity *H_a_*, light environment *PPF* and stem water potential $\Psi_{Stem}$. It outputs the resulting dynamics of the dry and fresh masses and of the concentration of the various compounds. During the simulation of a mango tree development, the fruit growth model is applied to each fruiting branch independently. A harvest date is determined for each fruit as the date when its sucrose concentration exceeds a threshold set at the average value of sucrose concentration in mango flesh cv. Cogshall at maturity in Reunion Island (Léchaudel et al., 2006).

Table 1. Main constant parameters used in the model

| Parameter (unit) | Equation | Value | References |
| --- | --- | --- | --- |
| Carbon assimilation by leaves | | | |
| *p_1_* (μmol CO_2_ m^-2^ s^-1^ g^-1^) | 2 | 3.85 ± 0.57 | Léchaudel et al. (2005) |
| p_2_ (μmol CO_2_ m^-2^ s^-1^) | 2 | 33.23 ± 11.91 |  |
| *p_3_* (dimensionless) | 1 | 0.483 ± 0.074 |  |
| *p_4_* (dimensionless) | 1 | 0.034 ± 0.007 |  |
| $P_{max}^{*}$ (μmol CO_2_ m^-2^ s^-1^) | 2 | 15.0 |  |
| Potential fruit growth | | | |
| *RGR_ini_* (dd^-1^) | 5 | 0.0105 ± 0.0003 | Léchaudel et al. (2005) |
| *GRC_fruit_* (g carbon g^-1^) | 5 | 0.04 ± 0.01 |  |
| *c_fruit_* (g carbon g^-1^) | 5 | 0.4239 ± 0.0048 |  |
| Transpiration | | | |
| ρ (cm d^-1^) | 6 | 5544.0 ± 141.6 | Léchaudel et al. (2007) |
| *H_f_* (dimensionless) | 6 | 0.996 |  |
| Plastic fruit growth | | | |
| a.L_f_ (g cm^-2^ MPa^-1^ d^-1^) | 8 | 0.3732 | Léchaudel et al. (2007) |
| h (MPa g^-1^) | 8 | 2.027 10-3 ± 3.6 10-5 |  |
| φ_max_ (MPa^-1^ j^-1^) | 13 | 0.414 |  |
| τ | 13 | 0.966 ± 0.071 |  |

**Reference**

**Fishman S. and Génard, M. 1998.** A biophysical model of fruit growth: simulation of seasonal and diurnal dynamics of mass. *Plant Cell Environment.* **21**:739-752.

**Léchaudel M., Génard M., Lescourret F., Urban L. and Jannoyer M., 2005.** Modeling effects of weather and source-sink relationships on mango fruit growth. *Tree Physiology* **25**: 583-597.

**Léchaudel M, Normand F, Génard M. 2006**. Predicting Harvest Date and Quality of Mango (cv ‘Cogshall’) Fruit According to Environmental Factors. *Proceedings of the VIII International Mango Symposium.* Ed. S.A. Oosthuyse. February 5-10. Sun City, South Africa.

**Léchaudel M., Vercambre G., Lescourret F., Normand F. and Génard M., 2007.** An analysis of elastic and plastic fruit growth of mango in response to various assimilate supplies. *Tree Physiology* **27**(2): 219-230.

**Lockart, J.A. 1965**. An analysis of irreversible plant cell elongation. *Journal of Theoretical Biology* **8**: 264-275.
